# Lipids as potential mediators linking body mass index to diabetes: evidence from a mediation analysis based on the NAGALA cohort

**DOI:** 10.1186/s12902-024-01594-5

**Published:** 2024-05-10

**Authors:** Song Lu, Qun Wang, Hengcheng Lu, Maobin Kuang, Min Zhang, Guotai Sheng, Yang Zou, Xiaoping Peng

**Affiliations:** 1 Department of Cardiology, The First Affiliated Hospital of Nanchang University, Jiangxi Medical College, Nanchang University, Nanchang, 330006 China; 2 Jiangxi Cardiovascular Research Institute, Jiangxi Provincial People’s Hospital, The First Affiliated Hospital of Nanchang Medical College, Nanchang, 330006 China; 3 Jiangxi Hypertension Research Institute, Nanchang, 330006 China

**Keywords:** Body mass index, Lipid parameters, Diabetes, Indirect effect, Mediation analyses

## Abstract

**Background:**

Body mass index (BMI) and lipid disorders are both known to be strongly associated with the development of diabetes, however, the indirect effect of lipid parameters in the BMI-related diabetes risk is currently unknown. This study aimed to investigate the mediating role of lipid parameters in the association of BMI with diabetes risk.

**Methods:**

We assessed the association of diabetes risk with BMI, as well as lipid parameters including high-density lipoprotein cholesterol(HDL-C), low-density lipoprotein cholesterol(LDL-C^F^ and LDL-C^S^), triglycerides(TG), total cholesterol(TC), remnant cholesterol(RC), non-HDL-C, and combined indices of lipid parameters with HDL-C (RC/HDL-C ratio, TG/HDL-C ratio, TC/HDL-C ratio, non-HDL/HDL-C ratio, LDL/HDL-C ratio) using data from 15,453 subjects in the NAGALA project. Mediation models were used to explore the mediating role of lipid parameters in the association of BMI with diabetes risk, and mediation percentages were calculated for quantifying the strength of the indirect effects. Finally, receiver operating characteristic curve (ROC) analysis was used to compare the accuracy of BMI and BMI combined with lipid parameters in predicting incident diabetes.

**Results:**

Multivariate regression models, adjusted for confounding factors, demonstrated robust associations of lipid parameters, BMI, with diabetes risk, with the exception of TC, LDL-C^F^, LDL-C^S^, and non-HDL-C. Mediation analysis showed that lipid parameters except TC, LDL-C^F^, LDL-C^S^, and Non-HDL-C were involved in and mediated the association of BMI with diabetes risk, with the largest mediation percentage being the RC/HDL-C ratio, which was as high as 40%; it is worth mentioning that HDL-C and HDL-C-related lipid ratio parameters also play an important mediating role in the association between BMI and diabetes, with the mediator proportion being greater than 30%. Finally, based on the ROC results, we found that the prediction performance of all lipid parameters in the current study except TC was significantly improved when combined with BMI.

**Conclusion:**

Our fresh findings suggested that lipid parameters partially mediated the association of BMI with diabetes risk; this result indicated that in the context of diabetes risk screening and disease management, it is important to not only monitor BMI but also pay attention to lipid parameters, particularly HDL-C and HDL-C-related lipid ratio parameters.

**Supplementary Information:**

The online version contains supplementary material available at 10.1186/s12902-024-01594-5.

## Background

Diabetes is a chronic metabolic disorder characterized by disturbances in blood glucose metabolism, leading to systemic involvement of multiple organs and systems [1]. The latest research forecasting models reported that the global prevalence of diabetes is 529 million in 2021, and driven by the obesity epidemic [2], a staggering 1.31 billion people are projected to live with diabetes globally by 2050 [3]. In addition, the incidence of diabetes is gradually showing a trend towards younger age [4], which poses a new and great challenge to global public health. Therefore, early identification of diabetes risk and early intervention based on risk factors is essential to reduce the incidence of diabetes as well as to slow down the disease progression.

It is well known that obesity is closely associated with the development of diabetes and is a key driver of the diabetes epidemic [2, 5, 6]. BMI is the most classic and simple measure of obesity [7] and an important obesity index for measuring the risk of developing diabetes [8–13]. To date, there is still no clear-cut mechanism linking BMI directly to the development of diabetes, and the main arguments around obesity leading to diabetes currently focus on insulin resistance (IR), impaired β-cell function, and metabolic damage from chronic inflammation [14–16]. Recently, an increasing number of studies have focused on the impact of obesity-related disorders of lipid metabolism on the pathogenesis of diabetes, and researchers have used metabolomics to identify a range of lipid markers of obesity-related diabetic risk, mainly including phospholipids and sphingolipids [17–21]. These findings suggest that lipids may play an important role in obesity-related diabetes risk. Considering the important value of BMI in risk assessment of diabetes and the potential impact of lipid metabolism on diabetes, it is important to further clarify the impact of lipid parameters on the association of BMI with diabetes risk in real-world studies, which could provide an important basis for clarifying the pathogenesis and the daily risk management of diabetes. To clarify the answer to this question, in the current study we used mediation analysis on data from 15,453 subjects of the NAGALA cohort to investigate lipid parameters that potentially mediate the link of BMI with diabetes risk, quantifying their contributions and identifying the most impactful lipids for diabetes risk management.

## Methods

### Data source and study population

**Fig. 1:**
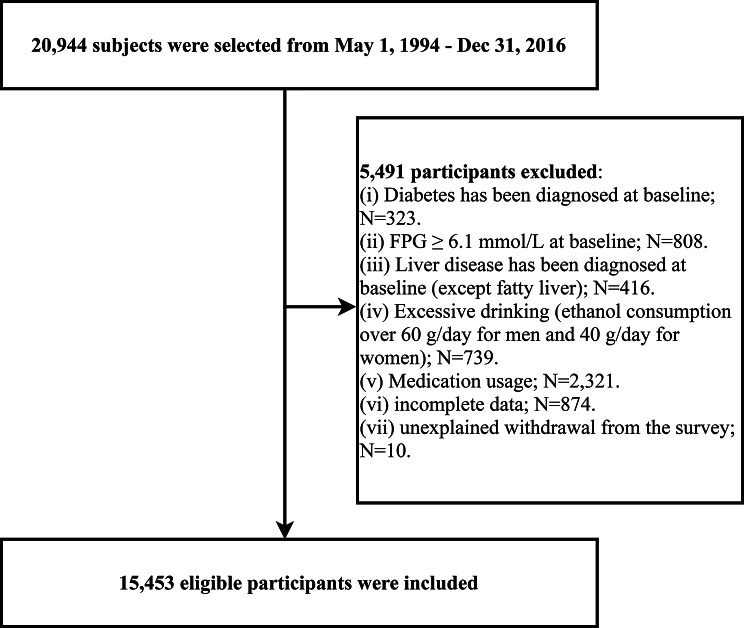
Flow chart of study participants

To elucidate the role of lipids in BMI-related diabetes risk, we extracted dataset from 20,944 participants in the NAGALA (1994–2016) project. The dataset was collected by Okamura’s team and stored in the Dryad public database (https://datadryad.org/stash/dataset/doi:10.5061/dryad.8q0p192). Based on Dryad’s Data Sharing Terms of Service, researchers can use publicly available data from the database to conduct in-depth analyses to explore new discoveries that will benefit academic progress. Detailed information about the NAGALA cohort study can be found in the previously published research [22]. According to the new research objectives, we further excluded the subjects with diabetes, impaired fasting glucose, liver disease, excessive drinking, incomplete data, using medicine at baseline, and withdraw survey with unknown reason, and finally, 15,453 subjects were included in the current study (Fig. 1). The NAGALA project has been authorized by the Ethics Committee of Murakami Memorial Hospital, and obtained informed consent from all subjects for their data usage [22]. This study was a secondary analysis of the NAGALA cohort study, which has been approved by the Ethics Committee of Jiangxi Province People’s Hospital. Furthermore, since the publicly available dataset has been anonymized, the Ethics Committee of Jiangxi Provincial People’s Hospital waived the need for informed consent from the subjects.

### Data collection, measurement, and calculation

**Fig. 2:**
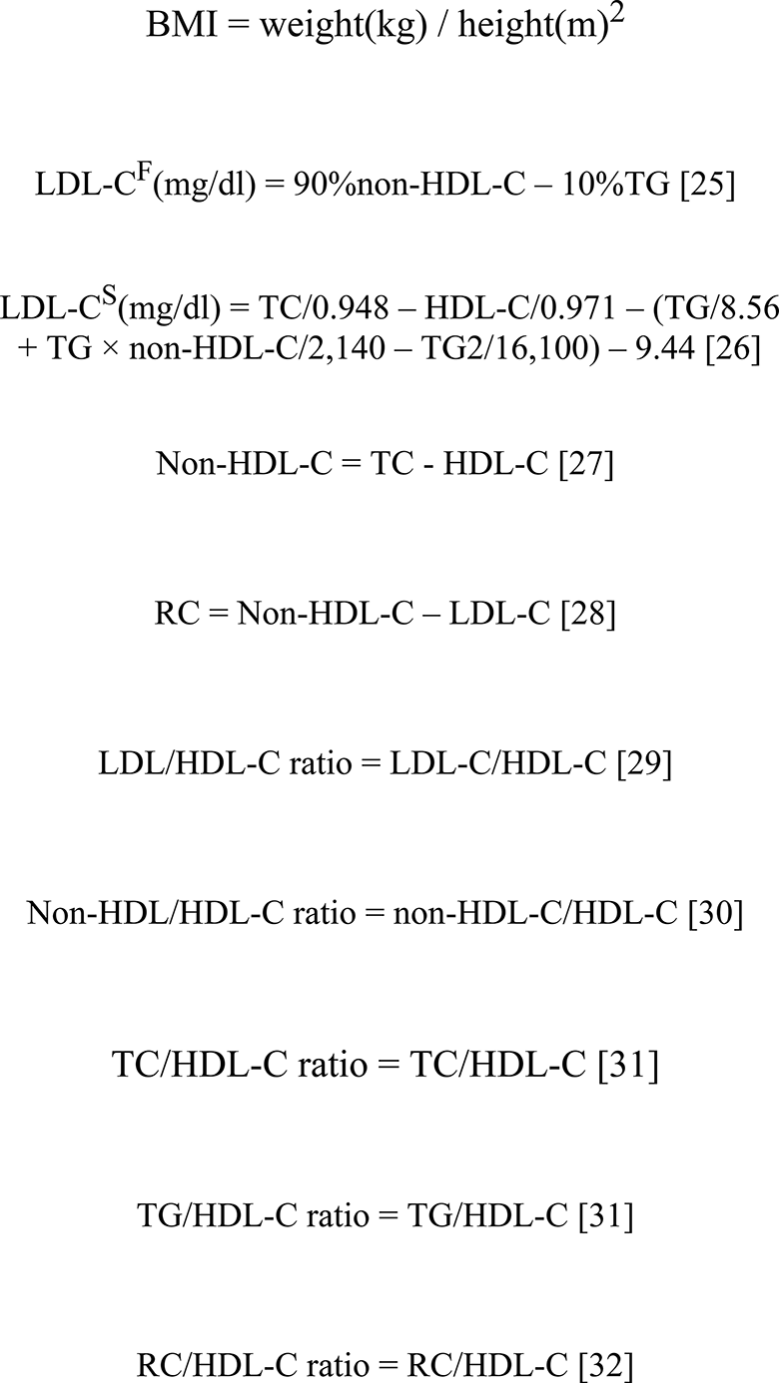
Formulas for calculating BMI and lipid parameters

All variables in the current study were contained in the NAGALA dataset [22], which were measured by the medical personnel using standardized methods and recorded in standardized questionnaires, including systolic/diastolic blood pressure (S/DBP), age, sex, weight, height, waist circumference (WC), TC, fasting plasma glucose (FPG), alanine aminotransferase (ALT), HDL-C, TG, gamma-glutamyl transferase (GGT), glycated hemoglobin (HbA1c), aspartate aminotransferase (AST), drinking status, smoking status, habit of exercise, and fatty liver. Among them, the blood biochemical indicators were measured and recorded by the automatic biochemical analyzer after all subjects fasting at least 8 h. Lifestyle factors were defined as following: (1) Drinking status was grouped according to the alcohol consumption in the past month, including non or little (< 40 g/w), light (40–139 g/w), moderate (140–279 g/w), and heavy (> 280 g/w) [23]. (2) Smoking status was grouped as non-smoking, former smoking, and current smoking. (3) Having exercise habits required engaging physical activity at least once a week. Moreover, fatty liver was diagnosed by gastroenterology experts based on abdominal ultrasound examination results of the subjects [24]. Based on baseline parameter information, we further calculated BMI and multiple lipid parameters, with the detailed calculation process presented in Fig. 2 [25–32]. The main results of the study on LDL-C are based on calculations using the Modified Friedewald Formula. To ensure the robustness of the results, we also used the Sampson formula to calculate LDL-C concentrations [26] (Fig. 2). In addition, to differentiate between the two methods of calculation, we referred to LDL-C results from the Modified Friedewald formula as LDL-C^F^ and those from the Sampson formula as LDL-C^S^.

### Diagnosis of diabetes

In the present study, the diagnosis of diabetes followed the criteria set by the American Diabetes Association [33], as follows: HbA1c ≥ 6.5% or FPG ≥ 7.0 mmol/L, along with self-reported diabetes.

### Statistical analysis

Subjects were grouped based on whether incident diabetes was diagnosed during follow-up [22]. We employed Marginal Structural Models to compute and quantify the magnitude of differences in baseline characteristics between diabetes and non-diabetes groups (prior to quantifying the differences, we performed BOX-COX transformations for skewed-distributed data), and differences greater than 10% were considered statistically significant [34, 35].

Prior to validating the mediation effect of lipid parameters in the association of BMI with diabetes risk, we computed the variance inflation factor for lipid parameters, BMI, and each covariate using linear regression equations, and covariates with variance inflation factor > 5 were considered collinear variables [36]. Based on the results of collinearity screening, we observed collinearity between all lipid parameters and weight and DBP, as well as between BMI and weight, DBP, and WC (Supplementary Tables 1–12); consequently, these variables (weight, DBP, and WC) will be excluded in subsequent multivariable regression models and mediation analyses.

We followed the approach recommended by Professor VanderWeele for conducting the mediation analysis [37, 38]. First, we employed a series of multivariable Cox regression models to examine the associations of lipid parameters/BMI with diabetes risk. In these models, we progressively adjusted for important demographic characteristics (height, age, sex), lifestyle factors (habit of exercise, drinking and smoking status), fatty liver, and metabolic factors related to blood pressure, blood glucose, and liver enzymes (SBP, GGT, FPG, ALT, HbA1c, AST) [1, 39, 40]. The strategy and process of progressive adjustment were conducted following the Strengthening the Reporting of Observational Studies in Epidemiology, and these steps were presented in the main analysis. Furthermore, when validating the association of BMI with diabetes risk, we conducted additional adjustments for the mediating variables (11 lipid parameters) and observed whether the association of BMI with diabetes weakened, to assess if the prerequisites for conducting a mediation analysis were met [37, 38]. Subsequently, we employed multiple linear regression to examine the association of BMI with lipid parameters, following the adjustment strategy outlined earlier [1, 41, 42]. Finally, we constructed mediation models to determine whether lipid parameters mediated the association of BMI with diabetes risk, and quantified the mediation effects of lipid parameters by calculating the mediation percentage, which is the ratio of the indirect effect to the total effect. To assess the significance of the mediation effects, we employed the Bootstrap sampling method with 1000 iterations. It is worth noting that the triglycerides glucose (TyG) index is calculated from parameters such as blood lipids and blood glucose. Considering that a large number of recent researches have shown that the TyG index is strongly associated with BMI and diabetes, in the current study we also examined the mediating role of the TyG index in the risk of BMI-related diabetes. Finally, ROC analysis was used to calculate the accuracy of BMI and BMI combined with lipid parameters in predicting incident diabetes, and the DeLong test was used to compare the area under the curve (AUC) among the models. All analyses were conducted using R version 3.4.3 and Empower(R) version 4.1. Two-tailed tests were employed, and statistical significance was set at *P* < 0.05.

## Results

### Characteristics of study subjects

A total of 15,453 subjects with a mean age of 43.7 ± 8.9 years were included in this study, and a total of 372 were diagnosed with new-onset diabetes. Table 1 shows the differences in the baseline characteristics of the study subjects grouped according to whether or not they were diagnosed with diabetes. We observed significant differences (standardized difference > 10%) in all baseline variables between the diabetes and non-diabetes groups. It is worth mentioning that the most substantial disparity between the two groups lay in the baseline glucose-related measures (FPG and HbA1c), with a standardized difference exceeding 100%. Furthermore, we observed that the BMI of diabetic participants was significantly higher than that of non-diabetic participants, with a standardized difference between the two groups reaching 86%. Lastly, it’s important to mention that in terms of lipid parameters, the standardized difference for the RC/HDL-C ratio was the largest (88%).

**Table 1:** Baseline characteristics of the study subjects with and without incident diabetes

	Non-diabetes	diabetes	Standardized difference (%)
No of subjects	15,080	373	
Sex			49 (39, 59)
Women	6947 (46.07%)	87 (23.32%)	
Men	8133 (53.93%)	286 (76.68%)	
Age, years	42.00 (37.00–50.00)	46.00 (41.00–53.00)	40 (30, 51)
Weight, kg	60.41 (11.48)	69.84 (13.32)	76 (66, 86)
Height, cm	1.65 (0.08)	1.67 (0.09) 1.68	19 (9, 29)
BMI, kg/m^2^	22.04 (3.07)	25.03 (3.82)	86 (76, 97)
WC, cm	76.25 (8.97)	85.08 (10.20)	92 (82, 102)
ALT, U/L	17.00 (13.00–23.00)	24.00 (18.00–39.00)	67 (56, 77)
AST, U/L	17.00 (14.00–21.00)	20.00 (16.00–26.00)	44 (34, 55)
GGT, U/L	15.00 (11.00–22.00)	24.00 (17.00–36.00)	47 (37, 58)
TC, mmol/L	5.12 (0.86)	5.43 (0.90)	35 (25, 46)
TG, mmol/L	0.72 (0.49–1.11)	1.21 (0.86–1.93)	73 (62, 83)
HDL-C, mmol/L	1.47 (0.40)	1.19 (0.33)	77 (66, 87)
LDL-C^F^, mmol/L	3.15 (2.63–3.69)	3.63 (3.09–4.14)	60 (50, 70)
LDL-C^S^, mmol/L	3.22 (2.70–3.79)	3.60 (3.08–4.20)	43 (33, 53)
Non-HDL-C, mmol/L	3.59 (3.00-4.23)	4.20 (3.57–4.82)	65 (55, 75)
RC, mmol/L	0.44 (0.36–0.53)	0.55 (0.46–0.67)	80 (70, 91)
TC/HDL-C ratio	3.50 (2.86–4.39)	4.71 (3.86–5.78)	87 (77, 97)
TG/HDL-C ratio	0.50 (0.30–0.89)	1.09 (0.64–1.93)	74 (63, 84)
LDL/HDL-C ratio	2.19 (1.64–2.96)	3.19 (2.50–4.11)	86 (75, 96)
Non-HDL/HDL-C ratio	2.50 (1.86–3.39)	3.71 (2.86–4.78)	87 (77, 97)
RC/HDL-C	0.30 (0.22–0.43)	0.48 (0.36–0.66)	88 (78, 98)
FPG, mmol/L	5.15 (0.41)	5.61 (0.36)	121 (111, 132)
HbA1c, %	5.16 (0.32)	5.53 (0.37)	107 (97, 118)
SBP, mmHg	114.31 (14.91)	122.03 (15.59)	51 (40, 61)
DBP, mmHg	71.44 (10.47)	77.18 (10.23)	55 (45, 66)
Habit of exercise	2655 (17.61%)	51 (13.67%)	11 (1, 21)
Fatty liver	2514 (16.67%)	223 (59.79%)	99 (89, 109)
Drinking status			21 (11, 31)
no or little	11,536 (76.50%)	266 (71.31%)	
light	1714 (11.37%)	40 (10.72%)	
moderate	1320 (8.75%)	37 (9.92%)	
heavy	510 (3.38%)	30 (8.04%)	
Smoking status			45 (35, 55)
non	8882 (58.90%)	145 (38.87%)	
former	2872 (19.05%)	77 (20.64%)	
current	3326 (22.06%)	151 (40.48%)	

Values were expressed as mean (SD) or medians (quartile interval) or n (%). Abbreviations: BMI: body mass index; WC: Waist circumference; ALT: alanine aminotransferase; AST: aspartate aminotransferase; GGT: gamma-glutamyl transferase; HDL-C: high-density lipoprotein cholesterol; TC: total cholesterol; TG: triglyceride; LDL-C: low density lipoprotein cholesterol; Non-HDL-C: non-high-density lipoprotein cholesterol; RC: remnant cholesterol; HbA1c: hemoglobin A1c; FPG: fasting plasma glucose; SBP: systolic blood pressure; DBP: Diastolic blood pressure

### Relationship of BMI with diabetes

After thorough adjustment for potential confounders, the current study also confirmed a significant positive correlation of BMI with the risk of diabetes (Table 2). In models 1 to 3, we progressively adjusted for all covariates except lipid parameters (mediating variables), and the hazard ratio (HR) for BMI-related diabetes risk was 1.096 in Model 3. Furthermore, we also included the lipid parameters (mediating variables) individually as covariates in the model (Table 2, models 4–14); the results revealed that except for the models where TC (Model 4: HR = 1.097) and LDL-C^F^ (Model 7: HR = 1.096) were incorporated as covariates for adjustment, other models indicated a weakening of the association of BMI with diabetes risk. These findings suggested that, apart from TC and LDL-C^F^, other lipid parameters might potentially mediate the association of BMI with diabetes risk.

**Table 2:** Relationship between BMI and incident diabetes

	HR (95%CI)	*P*-value
Model 1	1.250 (1.215, 1.286)	< 0.001
Model 2	1.153(1.115, 1.193)	< 0.001
Model 3	1.096 (1.057, 1.135)	< 0.001
Model 4	1.097 (1.059, 1.137)	< 0.001
Model 5	1.093 (1.054, 1.133)	< 0.001
Model 6	1.088(1.049, 1.128)	< 0.001
Model 7	1.096(1.058, 1.136)	< 0.001
Model 8	1.096 (1.057, 1.136)	< 0.001
Model 9	1.093 (1.054, 1.133)	< 0.001
Model 10	1.089 (1.050, 1.129)	< 0.001
Model 11	1.091 (1.053, 1.131)	< 0.001
Model 12	1.089 (1.051, 1.130)	< 0.001
Model 13	1.089 (1.050, 1.129)	< 0.001
Model 14	1.088 (1.049, 1.129)	< 0.001

Abbreviations: HR: Hazard ratios; CI: confidence interval; other abbreviations as in Table ​1

Model 1 adjusted sex, age, height, SBP

Model 2 adjusted model 1 + Fatty liver, habit of exercise, smoking status and drinking status

Model 3 adjusted model 2 + ALT, AST, GGT, FPG and HbA1c

Model 4 adjusted model 3 + TC; Model 5 adjusted model 3 + TG; Model 6 adjusted model 3 + HDL-C; Model 7 adjusted model 3 + LDL-C; Model 8 adjusted model 3 + Non-HDL-C; Model 9 adjusted model 3 + RC; Model 10 adjusted model 3 + TC/HDL-C ratio; Model 11 adjusted model 3 + TG/HDL-C ratio; Model 12 adjusted model 3 + LDL/HDL-C ratio; Model 13 adjusted model 3 + non-HDL/HDL-C ratio; Model 14 adjusted model 3 + RC/HDL-C ratio

Models 4–14 show the correlation between BMI and diabetes when lipid parameters are included in the regression model

### Relationship of lipid parameters with incident diabetes

We proceeded to run three multivariate Cox regression models to validate the associations of lipid parameters with diabetes risk (Table 3). It can be observed that in Model I, all lipid parameters were associated with diabetes risk; among them, apart from HDL-C which exhibited a negative correlation with diabetes, the rest of the lipid parameters showed a positive correlation with diabetes. However, in Models II and III, with further adjustments for demographic characteristics, lifestyle factors, fatty liver, and blood pressure and glucose enzyme metabolism factors, we observed that the associations between TC, LDL-C^F^, LDL-C^S^, and non-HDL-C with diabetes risk disappeared, which suggested that TC, LDL-C^F^, LDL-C^S^, and non-HDL-C may not be the mediating factors in the association of BMI with diabetes risk. Additionally, it’s important to note that among all lipid parameters, RC and lipid ratios such as RC/HDL-C exhibited the highest level of association with diabetes risk (RC: HR = 2.23; RC/HDL-C ratio: HR = 2.67).

**Table 3:** Relationship between lipid parameters and incident diabetes

	HR (95%CI)			
	Model I	Model II	Model III	
TC	1.29 (1.15, 1.45)	1.16 (1.03, 1.31)	0.94 (0.83, 1.07)	
TG	1.61 (1.48, 1.75)	1.39 (1.25, 1.54)	1.22 (1.08, 1.37)	
HDL-C	0.17 (0.12, 0.25)	0.37 (0.25, 0.54)	0.47 (0.32, 0.70)	
LDL-C^F^	1.58 (1.40, 1.79)	1.29 (1.13, 1.49)	1.00 (0.87, 1.16)	
LDL-C^S^	1.48 (1.34, 1.64)	1.23 (1.11, 1.27)	1.02 (0.91 1.14)	
Non-HDL-C	1.55 (1.39, 1.72)	1.29 (1.14, 1.45)	1.02 (0.91, 1.16)	
RC	34.45 (18.54, 64.03)	9.11 (4.45, 18.66)	2.23 (1.05, 4.71)	
TC/HDL-C ratio	1.53 (1.43, 1.64)	1.29 (1.20, 1.40)	1.15 (1.06, 1.25)	
TG/HDL-C ratio	1.39 (1.32, 1.46)	1.29 (1.20, 1.39)	1.20 (1.10, 1.31)	
LDL/HDL-C ratio	1.63 (1.51, 1.77)	1.34 (1.22, 1.47)	1.17 (1.06, 1.29)	
Non-HDL/HDL-C ratio	1.53 (1.43, 1.64)	1.29 (1.20, 1.40)	1.15 (1.06, 1.25)	
RC/HDL-C ratio	11.45 (8.09, 16.21)	5.12 (3.27, 8.02)	2.67 (1.64, 4.35)	

Abbreviations: Hazard ratios; CI: confidence interval; other abbreviations as in Table 1

Model I adjusted sex, age, height, SBP

Model II adjusted model I + Fatty liver, habit of exercise, smoking status and drinking status

Model III adjusted model II + ALT, AST, GGT, FPG and HbA1c

### Relationship of BMI with lipid parameters

Table 4 presents the results of the correlation analysis of BMI with lipid parameters. In linear regression, after thorough adjustment for confounding factors, we found significant associations between all lipid parameters and BMI, and except for HDL-C which exhibited a negative correlation with BMI (β=-2.47), the rest of the lipid parameters showed significant positive correlations with BMI. Additionally, it’s worth mentioning that the association of TC/HDL-C ratio with BMI was the strongest (β = 4.19).

**Table 4:** Association of BMI with lipid parameters

	β (95%CI)			
	Model I	Model II	Model III	
TC	1.78 (1.59, 1.96)	1.13 (0.96, 1.31)	0.79 (0.62, 0.97)	
TG	1.47 (1.40, 1.55)	0.97 (0.89, 1.04)	0.81 (0.74, 0.88)	
HDL-C	-3.51 (-3.68, -3.35)	-2.63 (-2.79, -2.47)	-2.47 (-2.63, -2.32)	
LDL-C^F^	2.24 (2.11, 2.36)	1.60 (1.48, 1.72)	1.36 (1.24, 1.48)	
LDL-C^S^	1.47 (1.37, 1.56)	1.08 (0.99, 1.71)	0.91 (0.82, 1.00)	
Non-HDL-C	2.22 (2.10, 2.34)	1.58 (1.46, 1.69)	1.34 (1.23, 1.45)	
RC	3.45 (3.29, 3.61)	2.40 (2.24, 2.55)	2.06 (1.90, 2.21)	
TC/HDL-C ratio	6.04 (5.81, 6.27)	4.63 (4.40, 4.86)	4.19 (3.96, 4.42)	
TG/HDL-C ratio	1.33 (1.27, 1.38)	0.94 (0.88, 1.00)	0.83 (0.77, 0.89)	
LDL/HDL-C ratio	2.60 (2.50, 2.70)	2.00 (1.90, 2.10)	1.81 (1.71, 1.91)	
Non-HDL/HDL-C ratio	2.62 (2.52, 2.72)	2.01 (1.91, 2.11)	1.82 (1.72, 1.92)	
RC/HDL-C ratio	2.26 (2.17, 2.34)	1.71 (1.62, 1.79)	1.54 (1.46, 1.63)	

Abbreviations: ***β*** : regression coefficient; CI: confidence interval; other abbreviations as in Table 1

Model I adjusted sex, age, height, SBP

Model II adjusted model I + Fatty liver, habit of exercise, smoking status and drinking status

Model III adjusted model II + ALT, AST, GGT, FPG and HbA1c

### Mediating effect of lipid parameters on the association of BMI with incident diabetes

**Fig. 3:**
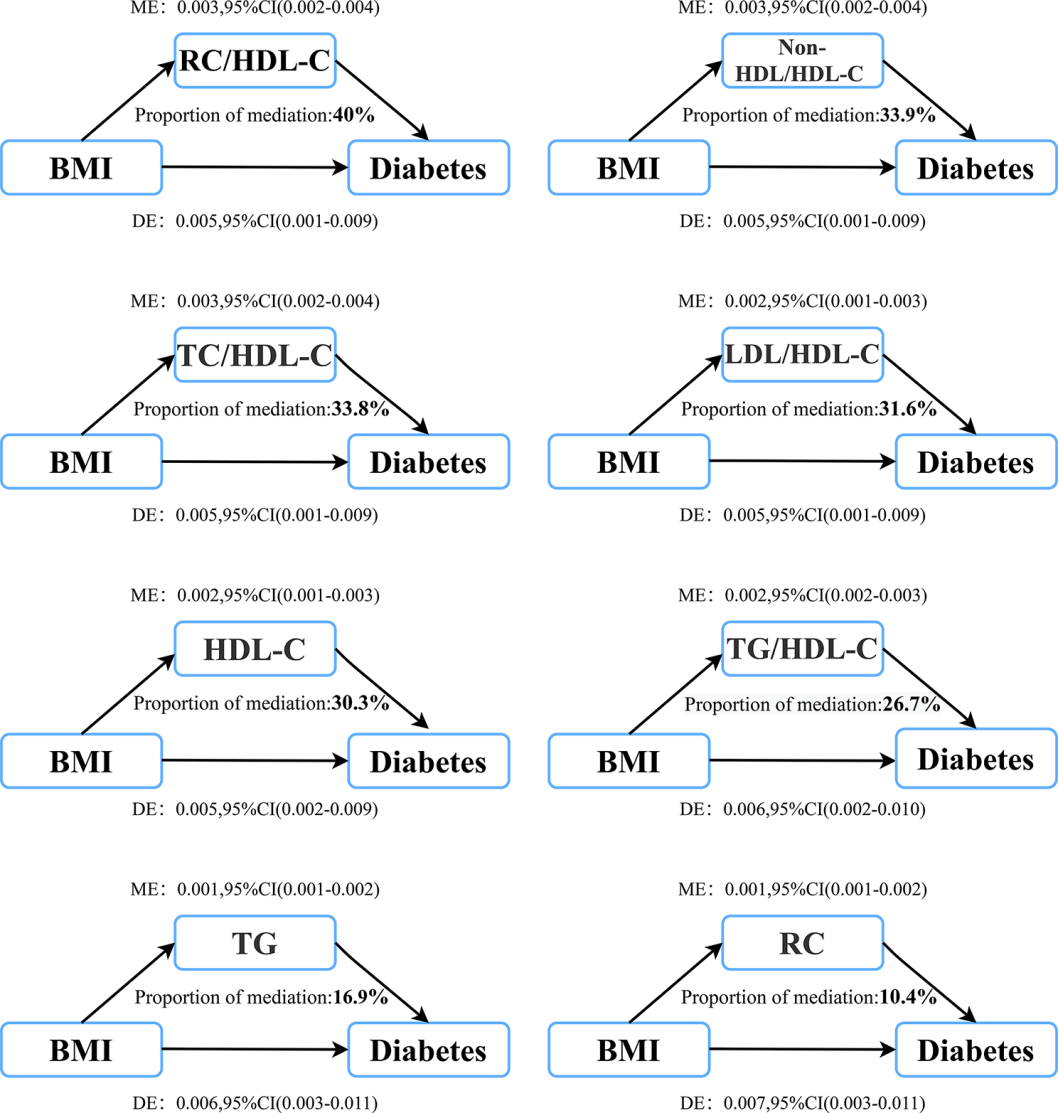
Lipid parameters mediation models of the relationship between BMI and incident diabetes. ME: Mediation effect; DE: Direct effect; BMI: Body mass index

Based on the results of the correlation analysis mentioned above, we proceeded to conduct further mediation analysis. Table 5; Fig. 3 present the results of the mediation analysis of lipid parameters in the association of BMI with the risk of diabetes incidence. The results revealed that, excluding TC, LDL-C^F^, LDL-C^S^, non-HDL-C, and TyG index the remaining 8 lipid parameters mediated the association of BMI with diabetes, with RC/HDL-C ratio having the most significant impact and a mediation percentage of 40%. Furthermore, it’s worth noting that the non-HDL/HDL-C ratio, TC/HDL-C ratio, LDL/HDL-C ratio, and HDL-C also exhibited mediation percentages exceeding 30%, establishing them as robust mediators in the association of BMI with diabetes risk; interestingly, unconventional lipid parameters tended to display higher mediation percentages compared to conventional ones.

**Table 5:** Mediation analysis for BMI and incident diabetes via lipid parameters in the whole population

Mediator	Total effect	Mediation effect	Direct effect	PM(%)	*p*-value of PM
TC	0.008 (0.004, 0.012)	-0.000 (-0.001, -0.000)	0.008 (0.004, 0.012)	-	-
TG	0.008 (0.004, 0.012)	0.001 (0.001, 0.002)	0.006 (0.003, 0.011)	16.9	< 0.001
HDL-C	0.008 (0.004, 0.012)	0.002 (0.001, 0.003)	0.005 (0.002, 0.009)	30.3	< 0.001
LDL-C^F^	0.008 (0.004, 0.012)	-0.000 (-0.001, 0.000)	0.008 (0.004, 0.012)	-	-
LDL-C^S^	0.008 (0.003, 0.013)	-0.001 (-0.001, 0.000)	0.009 (0.003, 0.014)	-	-
Non-HDL-C	0.008 (0.004, 0.012)	-0.000 (-0.001, 0.000)	0.008 (0.004, 0.012)	-	-
RC	0.008 (0.004, 0.012)	0.001 (0.000, 0.002)	0.007 (0.003, 0.011)	10.4	0.022
TC/HDL-C ratio	0.008 (0.004, 0.012)	0.003 (0.002, 0.004)	0.005 (0.001, 0.009)	33.8	< 0.001
TG/HDL-C ratio	0.008 (0.004, 0.012)	0.002 (0.002, 0.003)	0.006 (0.002, 0.010)	26.7	< 0.001
LDL/HDL-C ratio	0.008 (0.004, 0.012)	0.002 (0.001, 0.003)	0.005 (0.001, 0.009)	31.6	< 0.001
Non-HDL/HDL-C ratio	0.008 (0.004, 0.012)	0.003 (0.002, 0.004)	0.005 (0.001, 0.009)	33.9	< 0.001
RC/HDL-C ratio	0.008 (0.004, 0.012)	0.003 (0.002, 0.004)	0.005 (0.001, 0.009)	40	< 0.001
TyG index	0.008 (0.003, 0.013)	0.001 (-0.001, 0.001)	0.007 (0.002, 0.012)	-	-

Abbreviations: PM: proportion mediate; other abbreviations as in Table ​1

Adjusting variables: sex, age, height, SBP, Fatty liver, habit of exercise, smoking status, drinking status, ALT, AST, GGT, FPG and HbA1c

### Area under the ROC curve, specificity, sensitivity, PPV, and NPV of BMI and BMI combined lipid parameters to predict incident diabetes

The results in Table 6 demonstrate that when combined with BMI, all lipid parameters resulted in higher AUC values compared to BMI alone. The combination of RC/HDL-C ratio and BMI had the highest AUC value of 0.7748. Moreover, the accuracy of identifying incident diabetes was significantly enhanced by incorporating BMI with all lipid parameters, except for TC + BMI (All DeLong test *p* < 0.05).

**Table 6:** Area under the ROC curve, Specificity, Sensitivity, PPV, and NPV of BMI and BMI combined lipid parameters to predict incident diabetes

	AUC	95%CI low	95%CI upp	Specificity	Sensitivity	PPV	NPV
BMI	0.7327	0.7069	0.7586	0.7184	0.6273	0.0522	0.9873
TC + BMI	0.7369	0.7111	0.7627	0.7471	0.6086	0.0562	0.9872
TG + BMI*	0.7655	0.7406	0.7904	0.7438	0.6273	0.0605	0.9891
HDL-C + BMI*	0.7631	0.7388	0.7874	0.6977	0.7131	0.0522	0.0551
LDL-C^F^+ BMI*	0.7468	0.7271	0.7719	0.7164	0.6702	0.0522	0.9877
LDL-C^S^+ BMI*	0.7378	0.7124	0.7632	0.6758	0.6836	0.0496	0.9886
Non-HDL-C + BMI*	0.7503	0.7254	0.7753	0.7192	0.6729	0.0560	0.9889
RC + BMI*	0.7655	0.7409	0.7901	0.7186	0.6997	0.0579	2.3902
TC/HDL-C ratio + BMI*	0.7705	0.7468	0.7943	0.7737	0.6354	0.0649	0.9885
TG/HDL-C ratio + BMI*	0.7666	0.7418	0.7915	0.6740	0.7319	0.0526	0.9903
LDL/HDL-C ratio + BMI*	0.7688	0.7450	0.7925	0.7729	0.6273	0.0640	0.9882
Non-HDL/HDL-C ratio + BMI*	0.7705	0.7486	0.7943	0.7737	0.6354	0.0649	0.9885
RC/HDL-C ratio + BMI*	0.7748	0.7509	0.7987	0.8133	0.6086	0.0746	0.9883

ROC, receiver-operating characteristic curve; AUC, area under the ROC curve; PPV, positive predictive value; NPV, negative predictive value; CI, confidence interval; Other abbreviations as in Table 1; *, DeLong test, *P* < 0.05 compared with BMI

## Discussion

In this study encompassing 15,453 participants, we identified that apart from TC, LDL-C, and non-HDL-C, lipid parameters including RC, HDL-C, TG, TG/HDL-C ratio, TC/HDL-C ratio, LDL/HDL-C ratio, non-HDL/HDL-C ratio and RC/HDL-C ratio played significant mediating roles in the association of BMI with diabetes risk. Among them, the RC/HDL-C ratio made the most substantial contribution to the association of BMI with diabetes risk, accounting for a substantial 40%. Furthermore, the non-HDL/HDL-C ratio, LDL/HDL-C ratio, TC/HDL-C ratio and HDL-C exhibited significant mediation percentages as well, at 33.9%, 31.6%, 33.8% and 30.3%, respectively.

With the advancement of economies and shifts in lifestyle habits, the prevalence of diabetes is sweeping across the globe at an alarming rate, with a notable trend towards affecting younger individuals with lower body weight [1, 2]. Abundant epidemiological evidence underscores that both BMI and lipid dysregulation are pivotal risk factors in the onset and progression of diabetes [5, 43, 44]. BMI, serving as a body measurement indicator reflecting overall obesity, is associated with an elevated risk of diabetes and demonstrates a dose-response relationship; in comparison to other obesity indices, BMI might be the optimal predictor of diabetes risk [8, 11, 45, 46]. The investigation of lipid parameters in relation to the risk of diabetes has emerged as a recent research focus, particularly concerning composite lipid parameters [27, 30, 31, 47, 48]. In the present analysis, we assessed the associations between 11 lipid parameters and diabetes, and the findings revealed that a majority of lipid parameters were closely linked to the risk of diabetes, with the RC/HDL-C ratio displaying the strongest association with diabetes incidence risk. Building upon this, our subsequent mediation analysis revealed that the RC/HDL-C ratio serves as the most robust mediating factor in the association of BMI with diabetes risk. It is worth noting that the current study did not find significant associations between TC, LDL-C, and non-HDL-C with diabetes risk, which aligned with some previous reports [48–51]. However, it should be mentioned that certain studies have also indicated that TC, LDL-C, and non-HDL-C can indeed reflect diabetes risk [52, 53]. Further research is required to elucidate the discrepancies in these study outcomes. In the context of this study, the lack of significant mediation effects of TC, LDL-C, and non-HDL-C in the association of BMI with diabetes risk might largely stem from the absence of an underlying link between TC, LDL-C, and non-HDL-C and diabetes risk, as this did not align with the prerequisite for mediation analysis. Notably, HDL-C and HDL-C-related ratio parameters play a very strong mediating role in BMI-related diabetes risk. In pathological states such as obesity and diabetes, HDL-C function and composition are remodeled, mainly in the form of increased serum glycosylated proteins and oxidized HDL, which in turn reduces the antioxidant and anti-inflammatory effects of HDL [54, 55]. Taken together with the current study results, HDL-C may be the most single important lipid parameter in BMI-associated diabetes risk.To our knowledge, this is the first study to dissect the intricate relationship of BMI, lipid parameters, with diabetes risk. The current study demonstrated that the majority of lipid parameters were involved in mediating the association of BMI with the risk of diabetes. Regarding the mechanisms through which lipid parameters mediated the association of BMI with diabetes, several findings based on fundamental research might offer partial explanations: Generally, as BMI increases, serum-free fatty acids tend to rise [56], and tissues like the liver, skeletal muscle, and pancreas accelerate the uptake and utilization of free fatty acids. However, when these tissues reach a compensatory limit in their uptake and utilization of fatty acids, excess lipids accumulate within cells. On one hand, this lipid toxicity exacerbates lipid accumulation in β-cells, ultimately leading to β-cell apoptosis [44]; on the other hand, excessive lipid accumulation induces secondary IR in tissues like the liver and skeletal muscle [57], ultimately culminating in diabetes development. Indeed, the inflammatory response in adipose tissue is also noteworthy. When fatty acid uptake by adipocytes increases, white adipose tissue secretes tumor necrosis factor-alpha (TNF-α), which, through intracellular and extracellular cascades, activates the NF-κB pathway, leading to oxidative stress in adipose tissue, inflammation in β-cells, and hindrance of insulin signal transduction, thereby exacerbating IR [57–60]. Furthermore, TNF-α inhibits the expression of the ADIPOQ gene, lowering serum adiponectin levels, which in turn weakens the oxidation process of free fatty acids in skeletal muscle and liver, leading to elevated serum free fatty acid concentrations and exacerbating metabolic disturbances in lipid metabolism [61, 62]. Simultaneously, the significant role of adiponectin in exerting anti-apoptotic effects on β-cells should not be overlooked [63]; decreased adiponectin levels could substantially increase the risk of diabetes. Hence, lipid toxicity, inflammation, and IR likely constitute vital pathological pathways that link lipid parameters with BMI-related diabetes risk.

The present study assessed the mediating role of lipid parameters in the association of BMI with diabetes risk, further ROC analyses showed that lipid parameters combined with HDL-C were significantly improved not only in predicting incident diabetes but also in mediating BMI-related diabetes risk. It is noteworthy that in the current analysis, we identified the RC/HDL-C ratio as the most valuable lipid parameter for assessing diabetes risk and mediating the association of BMI with diabetes risk; therefore, we recommend placing emphasis on the simple yet efficient indicator of RC/HDL-C ratio in diabetes risk screening. Additionally, based on the results of the mediation analysis, it’s essential to emphasize that incorporating lipid levels into the management of diabetes risk, alongside weight management, could be an effective measure to reduce the incidence of diabetes. Existing evidence also suggested that addressing a single risk factor may prove inadequate for achieving favorable clinical outcomes in the context of complex metabolic disorders [63–66]. Therefore, implementing multifactorial management targeting the risk factors for diabetes could be a more effective approach, and this viewpoint is supported by a range of completed and ongoing clinical studies. Previous studies have shown that intensive interventions targeting multiple factors, including blood glucose, lipid levels, and blood pressure, among high-risk individuals, have resulted in a significant reduction in the risk of diabetes and related complications [66–69]. Among them, the Steno-2 cohort study demonstrated that for diabetic patients, the importance of controlling blood lipids might even surpass that of controlling blood glucose [69], which underscored the significance of lipid interventions in comprehensive, multifactorial management of diabetes. Dietary intervention is also a crucial measure in comprehensive diabetes management. Researches indicated that daily intake of 15–35 g of fiber-rich foods and consumption of polyunsaturated fatty acids (omega-3 fatty acids) can significantly improve blood glucose and lipid metabolism in diabetic patients and high-risk individuals, while also aiding in weight reduction [70, 71]. These findings offer valuable insights for diabetes prevention and management. Monitoring diabetes risk factors and relying on metabolic control to reduce diabetes risk and incidence rates might be a more efficient approach to curbing the diabetes epidemic.

### Study strengths and limitations

Our strength lies in quantifying the effects of 8 lipid parameters in the association of BMI with diabetes risk through mediation analysis, thus introducing novel perspectives into the biological mechanisms underlying the link between BMI and diabetes risk. Furthermore, these newfound discoveries offer fresh evidence and viewpoints for diabetes prevention and management, holding significant clinical implications.

Similarly, there are certain limitations of our study that should be acknowledged. (1) Our study found that lipid parameters only partially mediated the association of BMI with diabetes risk, indicating the existence of several unknown potential mediators that necessitate further research for exploration; furthermore, the specific mechanisms by which lipid parameters mediate the association of BMI with diabetes risk also require additional foundational research for validation. (2) Despite controlling for multiple covariates during our study, there remain unmeasured confounding factors that could potentially lead to residual confounding. (3) Some studies have indicated that the use of lipid-lowering drugs (statins) might increase the risk of diabetes [72], and they can also influence cholesterol and lipid metabolism. However, since the original study excluded subjects using such medications [22], we couldn’t assess the impact of lipid-lowering drugs on our study results and hope that future research could address this issue. (4) As our participants were solely drawn from the general population in Japan, further validation is required to assess the applicability of the current study’s findings to other ethnic groups.

## Conclusion

This study discovered that the majority of lipid parameters mediated the association of BMI with diabetes risk. It is noteworthy that the impact of the RC/HDL-C ratio was the most significant, mediating 40% of the BMI-related diabetes risk. These findings offered new insights into the prevention and treatment of diabetes, and focusing on HDL-C and HDL-C-related lipid ratio parameters may be an essential measure in the comprehensive management of BMI-related diabetes.

### Electronic supplementary material

Below is the link to the electronic supplementary material.

Supplementary Material 1

## Data Availability

The datasets analysed during the current study are available in the Dryad repository. [https://datadryad.org/stash/dataset/doi:10.5061/dryad.8q0p192].

## Abbreviations

BMI: Body mass index
GGT: gamma-glutamyl transferase
TNF-α: tumor necrosis factor-alpha
HDL-C: high-density lipoprotein cholesterol
HR: hazard ratio
DBP: diastolic blood pressure
FPG: fasting plasma glucose
ALT: alanine aminotransferase
LDL-C: low-density lipoprotein cholesterol
IR: insulin resistance
SBP: systolic blood pressure
AST: aspartate aminotransferase
WC: waist circumference
TG: triglyceride
HbA1c: glycated hemoglobin A1c
RC: remnant cholesterol
TC: total cholesterol
