# Supplementary material for: Lipids as potential mediators linking body mass index to diabetes: evidence from a mediation analysis based on the NAGALA cohort
Source: BMC Endocr Disord. 2024 May 10;24:66. doi: 10.1186/s12902-024-01594-5 (PMC11083816; doi:10.1186/s12902-024-01594-5)
Supplement: Supplementary file 1 — Supplementary Material 1 [file 12902_2024_1594_MOESM1_ESM.docx]

Supplementary Table 1: Collinearity diagnostics steps of TC with other covariates.

|  | Variance inflation factor | | |
| --- | --- | --- | --- |
|  | Step 1 | Step 2 | Step 3 |
| TC | 1.2 | 1.2 | 1.2 |
| Sex | 3.1 | 3.1 | 3.1 |
| Age | 1.4 | 1.3 | 1.3 |
| Weight | 8.9 | NA | NA |
| Height | 3.6 | 2.5 | 2.5 |
| WC | 5.8 | 2 | 2 |
| ALT | 4.2 | 4.1 | 4.1 |
| AST | 3.3 | 3.3 | 3.3 |
| GGT | 1.5 | 1.5 | 1.5 |
| FPG | 1.5 | 1.5 | 1.5 |
| HbA1c | 1.3 | 1.3 | 1.3 |
| SBP | 5.6 | 5.6 | 1.4 |
| DBP | 5.7 | 5.7 | NA |
| Habit of exercise | 1 | 1 | 1 |
| Fatty liver | 1.5 | 1.5 | 1.5 |
| Drinking status | 1.3 | 1.3 | 1.3 |
| Smoking status | 1.4 | 1.4 | 1.4 |

Abbreviations as in Table ​1.

Note: Variance inflation factor = 1/(1-R^2^).

Supplementary Table 2: Collinearity diagnostics steps of TG with other covariates.

|  | Variance inflation factor | | |
| --- | --- | --- | --- |
|  | Step 1 | Step 2 | Step 3 |
| TG | 1.4 | 1.4 | 1.4 |
| Sex | 3.1 | 3.1 | 3.1 |
| Age | 1.3 | 1.3 | 1.2 |
| Weight | 8.9 | NA | NA |
| Height | 3.5 | 2.5 | 2.5 |
| WC | 5.8 | 2 | 2 |
| ALT | 4.1 | 4.1 | 4.1 |
| AST | 3.3 | 3.3 | 3.3 |
| GGT | 1.5 | 1.5 | 1.5 |
| FPG | 1.5 | 1.5 | 1.5 |
| HbA1c | 1.3 | 1.3 | 1.3 |
| SBP | 5.6 | 5.6 | 1.4 |
| DBP | 5.7 | 5.7 | NA |
| Habit of exercise | 1 | 1 | 1 |
| Fatty liver | 1.6 | 1.5 | 1.5 |
| Drinking status | 1.3 | 1.3 | 1.3 |
| Smoking status | 1.4 | 1.4 | 1.4 |

Abbreviations as in Table ​1.

Note: Variance inflation factor = 1/(1-R^2^).

Supplementary Table 3: Collinearity diagnostics steps of HDL-C with other covariates.

|  | Variance inflation factor | | |
| --- | --- | --- | --- |
|  | Step 1 | Step 2 | Step 3 |
| HDL-C | 1.5 | 1.5 | 1.5 |
| Sex | 3.2 | 3.2 | 3.2 |
| Age | 1.3 | 1.3 | 1.2 |
| Weight | 8.9 | NA | NA |
| Height | 3.6 | 2.5 | 2.5 |
| WC | 5.8 | 2.1 | 2.1 |
| ALT | 4.1 | 4.1 | 4.1 |
| AST | 3.3 | 3.3 | 3.3 |
| GGT | 1.5 | 1.5 | 1.4 |
| FPG | 1.5 | 1.5 | 1.5 |
| HbA1c | 1.3 | 1.3 | 1.3 |
| SBP | 5.6 | 5.6 | 1.4 |
| DBP | 5.7 | 5.7 | NA |
| Habit of exercise | 1 | 1 | 1 |
| Fatty liver | 1.5 | 1.5 | 1.5 |
| Drinking status | 1.3 | 1.3 | 1.3 |
| Smoking status | 1.4 | 1.4 | 1.4 |

Abbreviations as in Table ​1.

Note: Variance inflation factor = 1/(1-R^2^).

Supplementary Table 4: Collinearity diagnostics steps of LDL-C with other covariates.

|  | Variance inflation factor | | |
| --- | --- | --- | --- |
|  | Step 1 | Step 2 | Step 3 |
| LDL-C | 1.3 | 1.3 | 1.3 |
| Sex | 3.1 | 3.1 | 3.1 |
| Age | 1.4 | 1.3 | 1.3 |
| Weight | 8.9 | NA | NA |
| Height | 3.6 | 2.5 | 2.5 |
| WC | 5.8 | 2 | 2 |
| ALT | 4.2 | 4.1 | 4.1 |
| AST | 3.3 | 3.3 | 3.3 |
| GGT | 1.5 | 1.5 | 1.5 |
| FPG | 1.5 | 1.5 | 1.5 |
| HbA1c | 1.3 | 1.3 | 1.3 |
| SBP | 5.6 | 5.6 | 1.4 |
| DBP | 5.7 | 5.7 | NA |
| Habit of exercise | 1 | 1 | 1 |
| Fatty liver | 1.5 | 1.5 | 1.5 |
| Drinking status | 1.3 | 1.3 | 1.3 |
| Smoking status | 1.4 | 1.4 | 1.4 |

Abbreviations as in Table ​1.

Note: Variance inflation factor = 1/(1-R^2^).

Supplementary Table 5: Collinearity diagnostics steps of Non-HDL-C with other covariates.

|  | Variance inflation factor | | |
| --- | --- | --- | --- |
|  | Step 1 | Step 2 | Step 3 |
| Non-HDL-C | 1.4 | 1.4 | 1.4 |
| Sex | 3.1 | 3.1 | 3.1 |
| Age | 1.4 | 1.3 | 1.3 |
| Weight | 8.9 | NA | NA |
| Height | 3.6 | 2.5 | 2.5 |
| WC | 5.8 | 2 | 2 |
| ALT | 4.2 | 4.1 | 4.1 |
| AST | 3.3 | 3.3 | 3.3 |
| GGT | 1.5 | 1.5 | 1.5 |
| FPG | 1.5 | 1.5 | 1.5 |
| HbA1c | 1.3 | 1.3 | 1.3 |
| SBP | 5.6 | 5.6 | 1.4 |
| DBP | 5.7 | 5.7 | NA |
| Habit of exercise | 1 | 1 | 1 |
| Fatty liver | 1.5 | 1.5 | 1.5 |
| Drinking status | 1.3 | 1.3 | 1.3 |
| Smoking status | 1.4 | 1.4 | 1.4 |

Abbreviations as in Table ​1.

Note: Variance inflation factor = 1/(1-R^2^).

Supplementary Table 6: Collinearity diagnostics steps of RC with other covariates.

|  | Variance inflation factor | | |
| --- | --- | --- | --- |
|  | Step 1 | Step 2 | Step 3 |
| RC | 1.5 | 1.5 | 1.5 |
| Sex | 3.1 | 3.1 | 3.1 |
| Age | 1.4 | 1.3 | 1.3 |
| Weight | 8.9 | NA | NA |
| Height | 3.6 | 2.5 | 2.5 |
| WC | 5.8 | 2 | 2 |
| ALT | 4.2 | 4.1 | 4.1 |
| AST | 3.3 | 3.3 | 3.3 |
| GGT | 1.5 | 1.5 | 1.5 |
| FPG | 1.5 | 1.5 | 1.5 |
| HbA1c | 1.3 | 1.3 | 1.3 |
| SBP | 5.6 | 5.6 | 1.4 |
| DBP | 5.7 | 5.7 | NA |
| Habit of exercise | 1 | 1 | 1 |
| Fatty liver | 1.6 | 1.5 | 1.5 |
| Drinking status | 1.3 | 1.3 | 1.3 |
| Smoking status | 1.4 | 1.4 | 1.4 |

Abbreviations as in Table ​1.

Note: Variance inflation factor = 1/(1-R^2^).

Supplementary Table 7: Collinearity diagnostics steps of TC/HDL-C ratio with other covariates.

|  | Variance inflation factor | | |
| --- | --- | --- | --- |
|  | Step 1 | Step 2 | Step 3 |
| TC/HDL-C ratio | 1.7 | 1.7 | 1.7 |
| Sex | 3.2 | 3.2 | 3.1 |
| Age | 1.3 | 1.3 | 1.3 |
| Weight | 8.9 | NA | NA |
| Height | 3.6 | 2.5 | 2.5 |
| WC | 5.8 | 2.1 | 2.1 |
| ALT | 4.2 | 4.1 | 4.1 |
| AST | 3.3 | 3.3 | 3.3 |
| GGT | 1.5 | 1.4 | 1.4 |
| FPG | 1.5 | 1.5 | 1.5 |
| HbA1c | 1.3 | 1.3 | 1.3 |
| SBP | 5.6 | 5.6 | 1.4 |
| DBP | 5.7 | 5.7 | NA |
| Habit of exercise | 1 | 1 | 1 |
| Fatty liver | 1.6 | 1.5 | 1.5 |
| Drinking status | 1.3 | 1.3 | 1.3 |
| Smoking status | 1.4 | 1.4 | 1.4 |

Abbreviations as in Table ​1.

Note: Variance inflation factor = 1/(1-R^2^).

Supplementary Table 8: Collinearity diagnostics steps of TG/HDL-C ratio with other covariates.

|  | Variance inflation factor | | |
| --- | --- | --- | --- |
|  | Step 1 | Step 2 | Step 3 |
| TG/HDL-C ratio | 1.4 | 1.4 | 1.4 |
| Sex | 3.1 | 3.1 | 3.1 |
| Age | 1.3 | 1.3 | 1.2 |
| Weight | 8.9 | NA | NA |
| Height | 3.5 | 2.5 | 2.5 |
| WC | 5.8 | 2 | 2 |
| ALT | 4.2 | 4.1 | 4.1 |
| AST | 3.3 | 3.3 | 3.3 |
| GGT | 1.5 | 1.5 | 1.5 |
| FPG | 1.5 | 1.5 | 1.5 |
| HbA1c | 1.3 | 1.3 | 1.3 |
| SBP | 5.6 | 5.6 | 1.4 |
| DBP | 5.7 | 5.7 | NA |
| Habit of exercise | 1 | 1 | 1 |
| Fatty liver | 1.6 | 1.5 | 1.5 |
| Drinking status | 1.3 | 1.3 | 1.3 |
| Smoking status | 1.4 | 1.4 | 1.4 |

Abbreviations as in Table ​1.

Note: Variance inflation factor = 1/(1-R^2^).

Supplementary Table 9: Collinearity diagnostics steps of LDL/HDL-C ratio with other covariates.

|  | Variance inflation factor | | |
| --- | --- | --- | --- |
|  | Step 1 | Step 2 | Step 3 |
| LDL/HDL-C ratio | 1.7 | 1.6 | 1.6 |
| Sex | 3.2 | 3.2 | 3.1 |
| Age | 1.4 | 1.3 | 1.3 |
| Weight | 8.9 | NA | NA |
| Height | 3.6 | 2.5 | 2.5 |
| WC | 5.8 | 2.1 | 2.1 |
| ALT | 4.2 | 4.1 | 4.1 |
| AST | 3.3 | 3.3 | 3.3 |
| GGT | 1.5 | 1.4 | 1.4 |
| FPG | 1.5 | 1.5 | 1.5 |
| HbA1c | 1.3 | 1.3 | 1.3 |
| SBP | 5.6 | 5.6 | 1.4 |
| DBP | 5.7 | 5.7 | NA |
| Habit of exercise | 1 | 1 | 1 |
| Fatty liver | 1.5 | 1.5 | 1.5 |
| Drinking status | 1.3 | 1.3 | 1.3 |
| Smoking status | 1.4 | 1.4 | 1.4 |

Abbreviations as in Table ​1.

Note: Variance inflation factor = 1/(1-R^2^).

Supplementary Table 10: Collinearity diagnostics steps of Non-HDL/HDL-C ratio with other covariates.

|  | Variance inflation factor | | |
| --- | --- | --- | --- |
|  | Step 1 | Step 2 | Step 3 |
| Non-HDL/HDL-C ratio | 1.7 | 1.7 | 1.7 |
| Sex | 3.2 | 3.2 | 3.1 |
| Age | 1.3 | 1.3 | 1.3 |
| Weight | 8.9 | NA | NA |
| Height | 3.6 | 2.5 | 2.5 |
| WC | 5.8 | 2.1 | 2.1 |
| ALT | 4.2 | 4.1 | 4.1 |
| AST | 3.3 | 3.3 | 3.3 |
| GGT | 1.5 | 1.4 | 1.4 |
| FPG | 1.5 | 1.5 | 1.5 |
| HbA1c | 1.3 | 1.3 | 1.3 |
| SBP | 5.6 | 5.6 | 1.4 |
| DBP | 5.7 | 5.7 | NA |
| Habit of exercise | 1 | 1 | 1 |
| Fatty liver | 1.6 | 1.5 | 1.5 |
| Drinking status | 1.3 | 1.3 | 1.3 |
| Smoking status | 1.4 | 1.4 | 1.4 |

Abbreviations as in Table ​1.

Note: Variance inflation factor = 1/(1-R^2^).

Supplementary Table 11: Collinearity diagnostics steps of RC/HDL-C ratio with other covariates.

|  | Variance inflation factor | | |
| --- | --- | --- | --- |
|  | Step 1 | Step 2 | Step 3 |
| RC /HDL-C ratio | 1.7 | 1.7 | 1.7 |
| Sex | 3.1 | 3.1 | 3.1 |
| Age | 1.3 | 1.3 | 1.3 |
| Weight | 8.9 | NA | NA |
| Height | 3.6 | 2.5 | 2.5 |
| WC | 5.8 | 2.1 | 2.1 |
| ALT | 4.2 | 4.1 | 4.1 |
| AST | 3.3 | 3.3 | 3.3 |
| GGT | 1.5 | 1.5 | 1.5 |
| FPG | 1.5 | 1.5 | 1.5 |
| HbA1c | 1.3 | 1.3 | 1.3 |
| SBP | 5.6 | 5.6 | 1.4 |
| DBP | 5.7 | 5.7 | NA |
| Habit of exercise | 1 | 1 | 1 |
| Fatty liver | 1.6 | 1.5 | 1.5 |
| Drinking status | 1.3 | 1.3 | 1.3 |
| Smoking status | 1.4 | 1.4 | 1.4 |

Abbreviations as in Table ​1.

Note: Variance inflation factor = 1/(1-R^2^).

Supplementary Table 12: Collinearity diagnostics steps of BMI with other covariates.

|  | Variance inflation factor | | | |
| --- | --- | --- | --- | --- |
|  | Step 1 | Step 2 | Step 3 | Step 4 |
| BMI | 90.5 | 5 | 1.7 | 1.7 |
| Sex | 3.1 | 3.1 | 3.1 | 3.1 |
| Age | 1.3 | 1.3 | 1.2 | 1.2 |
| Weight | 159.5 | NA | NA | NA |
| Height | 49.2 | 2.8 | 2.3 | 2.3 |
| WC | 6 | 5.9 | NA | NA |
| ALT | 4.1 | 4.1 | 4.1 | 4.1 |
| AST | 3.3 | 3.3 | 3.3 | 3.3 |
| GGT | 1.5 | 1.5 | 1.4 | 1.4 |
| FPG | 1.5 | 1.5 | 1.5 | 1.5 |
| HbA1c | 1.3 | 1.3 | 1.3 | 1.3 |
| SBP | 5.6 | 5.6 | 5.6 | 1.4 |
| DBP | 5.7 | 5.7 | 5.7 | NA |
| Habit of exercise | 1 | 1 | 1 | 1 |
| Fatty liver | 1.5 | 1.5 | 1.5 | 1.5 |
| Drinking status | 1.3 | 1.3 | 1.3 | 1.3 |
| Smoking status | 1.4 | 1.4 | 1.4 | 1.4 |

Abbreviations as in Table ​1.

Note: Variance inflation factor = 1/(1-R^2^).
